# The complete mitochondrial genome of *Microhyla butleri* (Amphibia, Anura, Microhylidae)

**DOI:** 10.1080/23802359.2016.1144107

**Published:** 2016-03-28

**Authors:** Shouhong Wang, Lusha Liu, Jianping Jiang

**Affiliations:** a Chengdu Institute of Biology, Chinese Academy of Sciences, Chengdu, China;; b University of Chinese Academy of Sciences, Beijing, China

**Keywords:** Microhylidae, *Microhyla butleri*, mitochondrial genome

## Abstract

The complete mitochondrial genome was determined from a *Microhyla butleri,* Microhylidae, *Microhyla*, which was collected from Shenzhen, China. The mitogenome was 16 714 bp in length, containing 13 protein-coding genes, two rRNA genes, 22 tRNA genes and a control region (D-loop). The base composition was 28.7% A, 29.5% T, 27.2% C and 14.6% G. The gene order and contents were identical to most amphibian mitogenome. Except *ND1* gene beginning with GTG and *COI* gene beginning with ATA, all other protein-coding genes began with ATG as start codon. Six protein-coding genes (*ND1*, *COII*, *ATP6*, *COIII*, *ND3* and *ND4*) ended with incomplete stop codon T. The 22 tRNA genes with the size ranging from 65 bp to 74 bp were interspersed along the whole genome. The D-loop region containing tandem repetition was 1334 bp in length and heavily biased to A + T nucleotides.

*Microhyla butleri* is known from central, southern and south-western China and it generally occurs in lowlands and at mid-altitudes up to about 220–1500 m (Frost 2015). We determined the complete mitogenome of this species. The sample of *M. butleri* was obtained from Shenzhen, Guangdong Province of China, with voucher number CIB20150408. Its muscle tissue sample was fixed with 95% ethanol and stored at −20 °C in the herpetological collection at Chengdu Institute of Biology, Chinese Academy of Sciences. The GenBank accession number is KT972718.

At present, four mitochondrial genome sequences of *Microhyla* species have been reported, including *M. pulchra, M. okinavensis, M. heymonsi* and *M. ornata* (GenBank accession numbers:NC_024547, AB303950, NC_006406 and NC_009422) (Zhang et al. 2005; Igawa et al. 2008; Wu et al. 2016). It is difficult to distinguish *M. ornata*, *M. heymonsi and M. butleri* from the appearance. In our study, we used 10 pairs of specific primers to amplify the mitochondrial complete genome, expecting to provide preliminary molecular data for further research.

The gene order and structure of *M. butleri* mitogenome is a circular molecule of 16 714 bp in size, containing 13 protein-coding genes, two rRNA genes, 22 tRNA genes and a control region. The base composition of the complete mitochondrial genome was 28.7% A, 29.5% T, 27.2% C and 14.6% G. The A + T base composition (58.2%) was higher than G + C (41.8%), agreeing with most vertebrates. The arrangement of genes in this mitogenome is identical to the general order of amphibian mitogenome (Chen et al. 2011). *ATP8* gene (165 bp) was the shortest and *ND5* was the longest gene (1791 bp) in 13 protein-coding genes. All protein-coding genes use ATG as start codon except *ND1* gene beginning with GTG and *COI* gene beginning with ATA. Except *ND6* gene and eight tRNA genes (*tRNA-Pro*, *tRNA-Gln*, *tRNA-Ala*, *tRNA-Asn*, *tRNA-Cys*, *tRNA-Tyr*, *tRNA-Ser* and *tRNA-Glu*) encoded on L-strand, remaining genes were coded on the heavy strand (H-strand). Six genes (*ND1*, *COII*, *ATP6*, *COIII*, *ND3* and *ND4L*) ended with an incomplete stop codon T which may be completed by posttranscriptional polyadenylation with poly A tail. The *12S rRNA* (941 bp) and *16S rRNA* (1579 bp) genes, located between *tRNA-Phe* and *tRNA-Leu* genes, were separated by *tRNA-Val* gene. The 22 tRNA genes with the size ranging from 65 bp to 74 bp were interspersed along the whole genome, and most of the tRNAs formed a colver-leaf structure except *tRNA-Ser*, which lost the T ψ C arm. The putative origin of L-strand replication (O_L_), with a length of 31 bp between the *tRNA-Asn* and *tRNA-Cys* genes, can fold into a stem loop of secondary structure, similar to other vertebrates (Boore 1999). The D-loop region, located between *Cytb* and *tRNA-Leu*, was 1333 bp in length. This region is heavily biased to A + T nucleotides (61.05%) and we found some repeated sequences in this region. For the whole mitogenome, there were 11 regions of gene overlap (ranging from 1 to 10 bp) and 11 intergenic spacer regions (ranging from 1 to 32 bp).

Based on the published mitogenome sequences of Microhylidae species, *Rana chensinensis* and *Rana kunyuensis* were selected as outgroup taxa to reconstruct a phylogenetic tree (Figure 1) using MEGA 6.0 (Tamura et al. 2013). Bayesian inference (BI) and maximum-likelihood (ML) analyses were used to reconstruct phylogenetic tree, which yielded the same topologies (Figure 1). The article will provide fundamental data for further investigating the phylogenetic study of the species.

**Figure 1.**
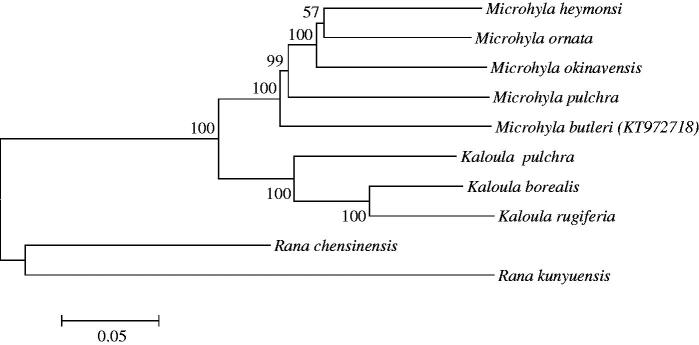
Neighbour-joining tree using MEGA 6.0 based on complete mitochondrial genomes. *Microhyla heymonsi* (NC_006406), *Microhyla ornata* (NC_009422), *Microhyla okinavensis* (AB303950), *Microhyla pulchra* (NC_024547), *Microhylabutleri* (KT972718), *Kaloula pulchra* (AY458595), *Kaloula borealis* (NC_020044), *Kaloula rugiferia* (KP682314), *Rana chensinensis*(NC_023529) and *Rana kunyuensis* (KF840516).
